# Long noncoding RNA transcriptome analysis reveals novel lncRNAs in *Morus alba* ‘Yu‐711’ response to drought stress

**DOI:** 10.1002/tpg2.20273

**Published:** 2022-10-26

**Authors:** Michael Ackah, Xin Jin, Qiaonan Zhang, Frank Kwarteng Amoako, Lei Wang, Thomas Attaribo, Mengdi Zhao, Feng Yuan, Richard Ansah Herman, Changyu Qiu, Qiang Lin, Zhi Yin, Weiguo Zhao

**Affiliations:** ^1^ Jiangsu Key Laboratory of Sericultural Biology and Biotechnology, School of Biotechnology Jiangsu Univ. of Science and Technology Zhenjiang 212100 China; ^2^ Institute of Plant Nutrition and Soil Science Kiel Univ. Hermann‐Rodewald‐Straße 2 Kiel 24118 Germany; ^3^ School of Agriculture C. K. Tedam Univ. of Technology and Applied Sciences Navrongo UK‐0215‐5321 Ghana; ^4^ Dep. of Materials Science and Engineering Suzhou Univ. of Science and Technology 99 Xuefu Road, Huqiu District Suzhou 215004 China; ^5^ Sericultural Research Institute Guangxi Zhuang Autonomous Region Nanning 530007 China; ^6^ Nanjing Univ. of Finance & Economics Nanjing 210023 China; ^7^ School of Food and Biological Engineering Jiangsu Univ. Zhenjiang Jiangsu 212013 People's Republic of China

## Abstract

Drought stress has been a key environmental factor affecting plant growth and development. The plant genome is capable of producing long noncoding RNAs (lncRNAs). To better understand white mulberry (*Morus alba* L.) drought response mechanism, we conducted a comparative transcriptome study comparing two treatments: drought‐stressed (EG) and well‐watered (CK) plants. A total of 674 differentially expressed lncRNAs (DElncRNAs) were identified. In addition, 782 differentially expressed messenger RNAs (DEmRNAs) were identified. We conducted Gene Ontology (GO) and KEGG enrichment analyses focusing on the differential lncRNAs *cis*‐target genes. The target genes of the DElncRNAs were most significantly involved in the biosynthesis of secondary metabolites. Gene regulatory networks of the target genes involving DElncRNAs–mRNAs–DEmRNAs and DElncRNA–miRNA–DEmRNA were constructed. In the DElncRNAs–DEmRNAs network, 30 DEmRNAs involved in the biosynthesis of secondary metabolites are collocated with 46 DElncRNAs. The interaction between DElncRNAs and candidate genes was identified using LncTar. In summary, quantitative real‐time polymerase chain reaction (qRT‐PCR) validated nine candidate genes and seven target lncRNAs including those identified by LncTar. We predicted that the DElncRNAs–DEmRNAs might recruit microRNAs (miRNAs) to interact with gene regulatory networks under the drought stress response in mulberry. The findings will contribute to our understanding of the regulatory functions of lncRNAs under drought stress and will shed new light on the mulberry–drought stress interactions.

## Core Ideas

- Mulberry response to drought stress reveals 674 differentially expressed lncRNAs.
- Target genes of the DElncRNAs were significantly enriched in the biosynthesis of secondary metabolites.
- Gene network reveals lncRNAs–miRNAs–mRNAs regulatory network involved in mulberry response to drought stress.
- Long noncoding RNAs participate in the host response to drought stress and interact with the target mRNAs.

**Abbreviations**
AS: alternative splicing
cDNA: complementary DNA
CK: well‐watered
CNCI: Coding‐Noncoding Index
CPC: Coding Potential Calculator
DEG: differentially expressed gene
DElncRNA: differentially expressed long noncoding RNA
DEmRNA: differentially expressed messenger RNA
EG: drought stressed
GO: Gene Ontology
lincRNA: intergenic lncRNA
lncRNA: long noncoding RNA
miRNA: microRNA
mRNA: messenger RNA
MXE: mutually exclusive exon
ncRNA: noncoding RNA
ndG: normalized deltaG
PCR: polymerase chain reaction
qRT‐PCR: quantitative real‐time polymerase chain reaction
SE: skipped exon
siRNA: small interfering RNA
rMAT: replicate multivariate analysis of transcript splicing

## 1 INTRODUCTION

Mulberry (*Morus* spp.), a significant food plant for silkworms (*Bombyx mori*), is widely cultivated worldwide in subtropical and temperate regions (Gai et al., 2018). As a result, the plant is subjected to various environmental challenges, including biotic and abiotic stresses, throughout its life cycle (Gai et al., 2018). These conditions severely hamper mulberry leaf quality and productivity. In addition, silkworm larvae and cocoon characteristics are affected when these stressed leaves are fed upon (Gai et al., 2018). It is, therefore, imperative to develop mulberry cultivars with increased environmental tolerance characteristics. Drought continues to be one of the most detrimental environmental stresses, as it impairs plant growth and productivity (Ackah et al., 2021). For plants, drought stress has a wide range of physical, morphological, and biochemical repercussions including decreased photosynthesis, interrupted cell growth and division, and decreased cell turgor (Guo et al., 2020). For example, the mulberry cultivar Yu‐711 demonstrates distinct anatomical, morphological, and agronomic characteristics under water stress (Ackah et al., 2021).

Ongoing global climate change has increased the amount of research devoted to elucidating how crops resist drought stress and improve their resistance level (Fracasso et al., 2016; Tan et al., 2020). It is well known that plant responses to drought include the production of second messenger substances like Ca^2+^, phosphatidylinositol, and reactive oxygen species, which cause an increase in intracellular calcium levels, initiating a cascade network of protein phosphorylation pathways (Huang et al., 2012). Thus, these target proteins play a direct role in cell protection or regulate the expression of several specific stress‐related genes via transcription factors, thus enhancing the plant's ability to withstand adverse conditions (Farooq et al., 2009). Evidence shows that most prior research on the molecular mechanisms underlying plant stress tolerance has concentrated on the roles of protein‐coding genes (Wang et al., 2015). However, many noncoding RNAs (ncRNAs) have been identified and implicated in various biological activities including plant responses to environmental stressors (Joshi et al., 2016; Zhao et al., 2016). Different classes of RNA, including microRNAs (miRNAs), small interfering RNAs (siRNAs), trans‐acting siRNAs, and long noncoding RNAs (lncRNAs), constitute the ncRNAs (Bazin et al., 2017). Plant miRNAs, usually (18–24 nucleotides), are a class of small ncRNAs found to control their targets through messenger RNA (mRNA) degradation or translational repression (Pasquinelli, 2012).

Long noncoding RNA is a form of an RNA transcript, longer than 200 nucleotides in length, and lacks or has limited ability to code for proteins (Kung et al., 2013; Quinn and Chang, 2016). However, it is widely evident that lncRNAs are significant regulators of transcription and gene expressions at the posttranscriptional level such as modulation of RNA stability and translation (Kornienko et al., 2013; Chen et al., 2016). Long noncoding RNAs are divided into three types based on their proximity to the protein‐coding gene: intergenic lncRNAs (lincRNAs; transcribed from within the gene's intergenic region), antisense (across from the protein‐coding gene's antisense strand), and intronic (derived from within the protein‐coding gene's intronic region) (Jin et al., 2020). Numerous lncRNAs have been identified in various model plants such as *Arabidopsis thaliana* (L.) Heynh., wheat (*Triticum aestivum* L.), maize (*Zea mays* L.), and rice (*Oryza sativa* L.) (Gai et al., 2018) because of advancements in next‐generation sequencing techniques and bioinformatics approaches, demonstrating that lncRNAs play a crucial role in a variety of biological processes in plant development and stress response (Zhang, Liao et al., 2014). In addition, abiotic stressors, like drought, have been shown to affect lncRNAs (Li et al., 2014). For instance, 664 lncRNAs in maize were sensitive to drought stress (Zhang, Han et al., 2014). In *Arabidopsis*, drought, cold, high‐salt, and abscisic acid treatments altered the differential expression of 1,832 lncRNAs (Liu et al., 2012). In some cases, it has been shown that lncRNA serves as a precursor for miRNAs and siRNAs, which play regulatory roles in the cell (Ma et al., 2014). In addition, several lncRNAs contain putative miRNA regulatory elements and may be targets of miRNAs or endogenous mimics of miRNAs and participate in the miRNA regulatory networks (Wu et al., 2013; Wang et al., 2015). Thus, plants have elaborate networks of regulatory interactions between multiple ncRNAs that can regulate various biological processes and reactions to environmental challenges, whether biotic or abiotic (Jalali et al., 2013; Zhang, Liao et al., 2014).

As far as we know, lncRNAs studied in mulberry are rare (Song et al., 2016; Gai et al., 2018). As a result, identifying and characterizing stress‐responsive lncRNAs and delineating their regulatory networks will help us better understand how mulberry responds to environmental challenges through gene regulation. This study performed genome‐wide transcriptome analysis on mulberry cultivar Yu‐711 under drought stress and without drought stress treatment. As a result, we discovered novel lncRNAs and protein‐coding mRNAs in Yu‐711 response to drought stress. In addition, the network of interactions between lncRNAs and protein‐coding mRNAs and their miRNAs precursors was also mapped out in this study. This study may improve our understanding of the complex gene expression regulation mechanisms in mulberry stress responses and provide a foundation for future research into the genes and ncRNAs that have considerable potential applicability in mulberry biotechnology.

## 2 MATERIALS AND METHODS

### 2.1 Plant materials and treatment

**FIGURE 1:**
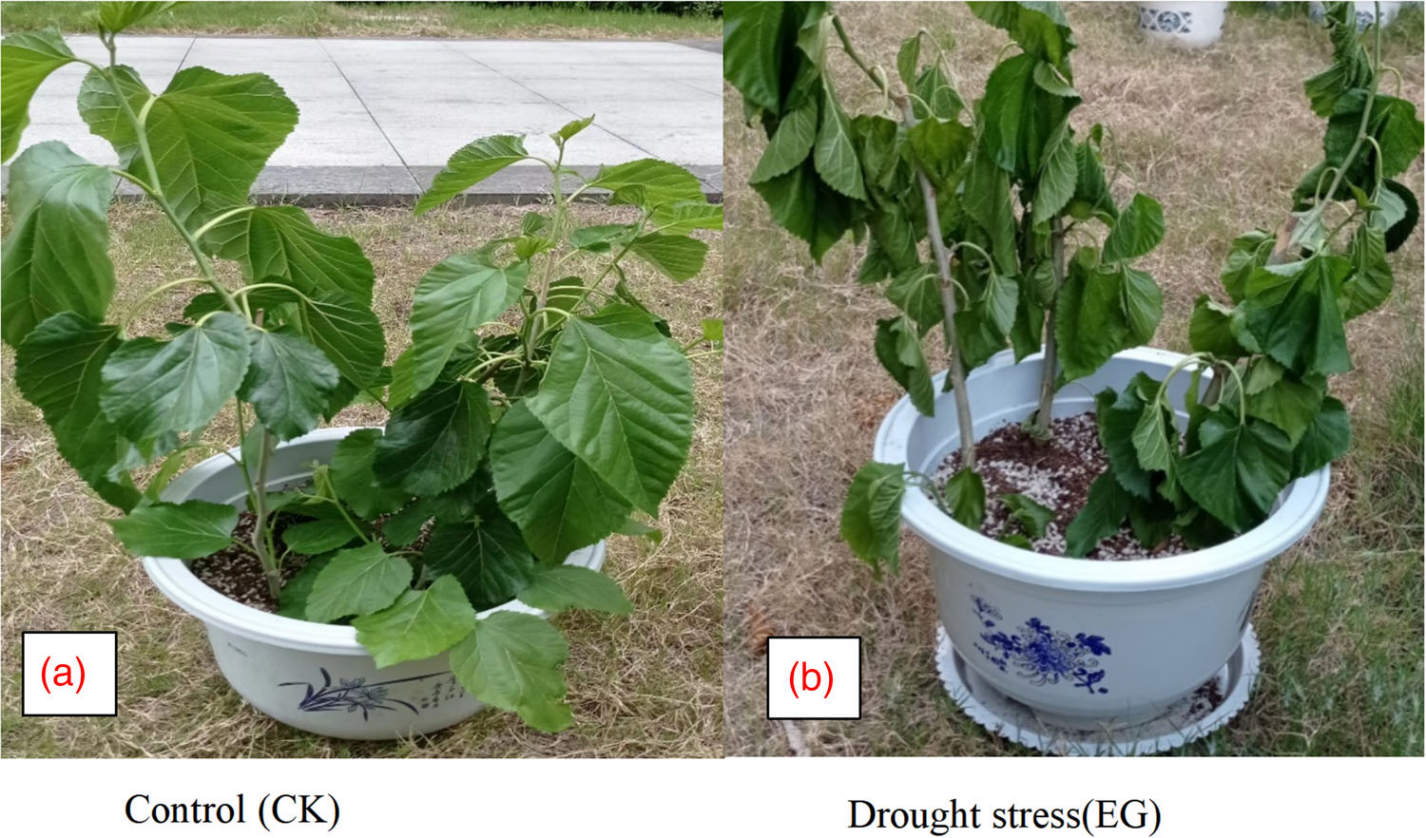
Physiological response of mulberry leaves affected by drought and the control treatment. (a) Mulberry plant under control treatment. (b) Mulberry plant under drought stress treatment at the 5‐d time points. EG, droughts stress treatment; CK, control treatment

All the mulberry (Yu‐711) seedlings were obtained from the National Mulberry GenBank at the Jiangsu University of Science and Technology, Zhenjiang, Jiangsu, China. Plants were grown in a greenhouse with a 14‐h light/10‐h dark photoperiod, at 25 °C day/20 °C night temperature and 70–80% relative humidity. The cultivation and drought stress treatments were performed according to Ackah et al. (2022). The leaves were sampled from the drought‐stress seedlings showing wilting points (symptoms apparent) and the control (Figure 1). The first three time points for sampling after the drought stress treatment was the first day (1 day), the third day (3 d), and the fifth day (5 d). The control and drought‐stressed plants were sampled simultaneously (midday). The primary leaf tissue samples were harvested and immediately frozen in liquid nitrogen and stored at −80 °C for further use. Mulberry leaves under drought stress (EG) and control (CK) treatments, with two replicates each, were used in this study to obtain a global view of drought‐induced lncRNAs and mRNAs.

**Core Ideas**

- Mulberry response to drought stress reveals 674 differentially expressed lncRNAs.
- Target genes of the DElncRNAs were significantly enriched in the biosynthesis of secondary metabolites.
- Gene network reveals lncRNAs–miRNAs–mRNAs regulatory network involved in mulberry response to drought stress.
- Long noncoding RNAs participate in the host response to drought stress and interact with the target mRNAs.

### 2.2 RNA extraction, strand‐specific cDNA library construction, and sequencing

Leaves from the EG and CK groups on the 5‐d time point (*n* = 4) were used for RNA extraction, complementary DNA (cDNA) library construction, and sequencing. Total RNA was extracted from the leaves sample using Trizol reagent (Invitrogen) according to the manufacturer's protocol. Residual genomic DNA was removed with DNase (TaKaRa). The RNA purity and concentration were then examined using a NanoPhotometer spectrophotometer (Invitrogen). The integrity of the total RNA was measured using the RNA Nano 6000 Assay Kit of the Bioanalyzer 2100 system (Bio‐Rad).

Strand‐specific cDNA libraries for the EG and CK groups were generated at Novogene Bioinformatics Technology Co., Ltd (Beijing, China) for lncRNA sequencing with two biological replicates. Briefly, the ribosomal RNA was depleted from total RNA using the ribosomal RNA Removal Kit following the manufacturer's instructions. The RNA was then fragmented into 250–300 bp fragments, and first‐strand cDNA was reverse‐transcribed using fragmented RNA and dNTPs (dATP, dUTP, dCTP, and dGTP). Then, the RNA was degraded using RNase H, and second‐strand cDNA was synthesized using DNA polymerase I and dNTPs. The remaining overhangs of double‐strand cDNA were converted into blunt ends via exonuclease and polymerase activities, after which adenylation of 3′ ends of DNA fragments and sequencing adaptors were ligated to the cDNA. The library fragments were purified with the AMPure XP system (Beckman Coulter) to preferentially select cDNA fragments 250–300 bp in length. Uridine digestion was performed using Uracil‐N‐Glycosylase (NEB) followed by the cDNA amplification using polymerase chain reaction (PCR). The library concentration was measured by the Qubit fluorometer and adjusted to 1 ng ul^−1^. Agilent 2100 Bioanalyzer (Agilent Technologies) was deployed to examine the insert size of the acquired library.

Furthermore, the accurate concentration of the cDNA library was examined again using quantitative PCR. After the library preparation, the libraries were subjected to Illumina sequencing using Hiseq X on the PE150 platform to generate reads with 150‐bp paired ends.

### 2.3 Quality control for raw data, mapping, and assembly

Raw data (raw reads) obtained after sequencing were firstly processed through in‐house Perl scripts because of the flexibility, power, and multipurpose functionality of using Perl scripts. In this step, clean data (clean reads) were obtained by removing the following reads: (a) reads with 5′ adapter, (b) reads without 3′ adapter or insert sequence, (c) reads with >10% N, (d) reads with >50% nucleotides with Qphred ≤ 20, and (e) reads with poly A/T/G/C. Furthermore, adapter trimming was performed to remove adapter sequences from the 3′ ends of reads. In addition, quality scores Q20 and Q30 and GC content of the clean data were calculated. Finally, all the downstream analyses were based on clean data with high quality. The high‐quality clean reads for each sample were first mapped to the mulberry reference genomic database (*M. notabilis* assembly, ASM41409v2) using HISAT2 v2.0.5 (Kim et al., 2015). The mapped reads result of each sample were then transferred to the StringTie v1.3.3 (Pertea et al., 2015) for transcript assembly.

### 2.4 Long noncoding RNA identification and classification

The assembled transcripts were merged using Cuffmerge software in the Cufflinks support package v2.2.1. Long noncoding RNAs were then identified from the assembled transcripts following steps: (a) removal of lowly expressed transcripts with fragments per kilobase of transcript per million mapped reads (FPKM) < 0.5, (b) removal of short transcripts <200 bp and <2 exons, (c) removal of the transcripts mapped within the 1‐kb flanking regions of an annotated gene using Cuffcompare in Cufflinks support package v2.2.1 (Ghosh and Chan, 2016), and (d) removal of the transcripts with protein‐coding capability using software Coding Potential Calculator v3.2.0 (CPC), Coding‐Noncoding Index (CNCI), and Pfam_scan (v1.3) (Mistry et al., 2007; Sun et al., 2013). Each software evaluated the coding potential of each transcript and compared the results to predetermined thresholds that discriminated between lncRNA and mRNA transcripts. The CPC and CNCI scoring thresholds were zero; transcripts with scores greater than zero were classified as mRNAs, while those below zero were classified as lncRNAs. Additionally, transcripts were classified as mRNAs or lncRNAs depending on whether they corresponded to the Pfam database (v1.3). A transcript was deemed as an ncRNA if the coding potential score of all the software was <0, suggesting the transcript had no protein‐coding capacity, otherwise, they were regarded as mRNAs. Novel lncRNAs were finally obtained and named following the HUGO Gene Nomenclature Committee rules (Wright, 2014). Finally, the lncRNA was obtained, and the characteristics of novel lncRNAs, such as transcript length, exon number, and open reading frame (ORF) were compared with mRNAs.

### 2.5 Quantification and differential expression analysis

The lncRNAs, mRNAs, and unclassified transcripts were quantified using StringTie v1.3.3 (Pertea et al., 2015), and FPKM was obtained. The edgeR software package v3.2.4 (Robinson et al., 2010) was used to identify and analyze the differentially expressed lncRNAs (DElncRNAs) and mRNAs (DEmRNAs). Differentially expressed mRNAs or DElncRNAs between EG and CK plants were selected using screening criteria of *p* < .05. The expression‐based sample clustering heat map, volcano map, and the principal component analysis were performed using the R package v3.2.4 (Robinson et al., 2010).

### 2.6 Analyses of GO and KEGG enrichment

Gene Ontology (GO) annotations were performed based on the GO database (http://www.geneontology.org/), and the GO enrichment analyses of DEmRNAs or the DElncRNA target genes were performed using the GOseq R package v3.3.2 (Young et al., 2010). The GO terms with corrected *p* value < .05 were considered significantly enriched, and the three terms—biological process, molecular function, and cellular component—were defined with default parameters. Furthermore, the metabolic pathway analysis of the DEmRNAs or the DElncRNA‐targeted genes was performed using the KEGG database (http://www.genome.jp/kegg). The KEGG pathways enrichment for the target genes was performed by the KOBAS software v2.0 (Kanehisa et al., 2017). The KEGG pathways with corrected *p* values < .05 were considered significant.

### 2.7 Prediction of DElncRNA–mRNA targets and DElncRNA–DEmRNA targets as miRNA precursors

We predicted the DElncRNA–mRNAs targets by using the collocation approach. We analyze the DElncRNA *cis*‐acting model by searching genes in a region covering 100 kb upstream to 100 kb downstream of individual DElncRNAs (Bao et al., 2019). The functional enrichment analysis (GO–KEGG) of target genes of differential lncRNAs was performed to predict the primary function of lncRNAs. This study did not perform the coexpression analysis because of the sample size. We also predicted the DElncRNA and DEmRNAs target miRNA precursors. We first downloaded matured miRNA sequences from the miRbase database (https://www.mirbase.org/; miRBase Release 21 June 2014) (Kozomara et al., 2019) using *Arabidopsis* miRNA as reference. Second, all the DElncRNA and DEmRNA targets were used to predict the putative miRNAs precursors using the online psRNATarget tool (https://www.zhaolab.org/psRNATarget/home) with the default parameters (Dai et al., 2018).

### 2.8 Competing endogenous RNA network analysis

LncRNAs and mRNA are competing endogenous RNAs that bind competitively to miRNAs and indirectly affect mRNA expression. We constructed competing endogenous RNA regulatory cascades, including DElncRNA–mRNA target and DElncRNA–DEmRNA–target miRNA using the online psRNATarget tool, and the networks were visualized using the Cytoscape v3.9.0 software (http://cytoscape.org).

### 2.9 LncRNA–mRNA interaction prediction

The tool LncTar (http://www.cuilab.cn/lnctar) is efficient for predicting lncRNAs target genes. The lncRNA and the target genes sequence were input to run the program. The normalized deltaG (ndG) cutoff was set to −0.05 out of the default value (−0.1). The ndG value was set as a threshold to serve as a cutoff point to determine if two molecules interacted with each other (Li et al., 2015). When the calculated ndG was less than the threshold, the gene was determined to be the target sequence of the lncRNAs.

### 2.10 Alternative splicing events detection and identification in mulberry plant response to drought stress

The rMATS software (http://rnaseq‐mats.sourceforge.net/index.html) (Shen et al., 2014) was used to identify alternative splicing (AS) events and the differential AS events between samples analyzed. The junction count‐only method was used in this study. The AS events were identified with a false discovery rate <0.05.

### 2.11 qRT‐PCR validation of the DElncRNA and DEmRNA results

To verify the DElncRNAs and DEmRNAs genes identified by high‐throughput sequencing, seven DElncRNAs and nine DEmRNAs were selected for quantitative real‐time polymerase chain reaction (qRT‐PCR) validation. We followed the protocol described in our previous study for the qRT‐PCR analysis (Ackah et al., 2022). In addition, fold changes in gene expression were estimated using the 2^−ΔΔCt^ method (Livak & Schmittgen, 2001). The primers used for the qRT‐PCRs and PCR validation are listed in Supplemental Table S8.

## 3 RESULTS

### 3.1 Physiological changes in mulberry Yu‐711 in response to drought stress and control treatment

Mulberry leaves underwent morphological alterations in response to drought stress, as illustrated in Figure 1. Therefore, physiological indicators such as relative water content and leaf lengths under drought stress and control were evaluated prior to transcriptome analysis to demonstrate how the drought stress progresses in the mulberry plant. The study technique and interpretation of the physiological parameter data can be found in Ackah et al. (2021).

### 3.2 Overview of the RNA‐seq results and quality control

The correlation of expression between the mulberry samples was examined, and the results showed that all the correlation coefficient (*R* ^2^) of samples was >.8 (Supplemental Figure. S1). The results show the reliability of our data samples. Table 1 shows the statistical data of the RNA sequencing (RNA‐seq) reads of each sample. A total of 332,924,366 raw reads were obtained through Illumina sequencing from the four libraries with an average of ∼83.23 million reads per sample. After quality‐control filtering, a total of 327,145,678 clean reads were obtained from the four libraries with average clean reads of ∼81.79 million per sample (Table 1). The quality scores Q20 and Q30 of the clean reads were 97.25 and 92.55% on average, respectively, indicating the quality of the clean reads obtained. Also, an average of 42.74% GC content was obtained from the sample's clean reads (Table 1). The bases distribution content along reads is shown in Supplemental Figure S2. Further analysis was carried out on the clean reads data.

**TABLE 1:** Raw reads sequencing data and quality control (QC)

Sample name	Raw reads	Clean reads	Raw bases (G)	Clean bases (G)	Error rate	Q20	Q30	GC content
					%			
CK1	83,338,066	81,866,870	12.5	12.28	0.03	97.17	92.3	43.12
CK2	83,683,996	81,783,646	12.55	12.27	0.03	97.29	92.79	42.67
E1	82,335,140	81,016,592	12.35	12.15	0.03	97.1	92.2	42.78
E2	83,567,164	82,478,570	12.54	12.37	0.03	97.44	92.89	42.37

First, clean reads were mapped to the *M. notabilis* reference genome using Hisat2 software (Kim et al., 2015). Sliding window density analysis showed that more than half of the transcripts were uniquely mapped to the reference genome (Table 2). A total of 208,033,333 clean reads from the four samples were uniquely mapped to the reference genome constituting an average of 64% mapped (Table 2). In addition, a total of 103,547,559 (average of 31.65%) of the clean reads were mapped to the sense strand, whereas 104,485,774 (averaging 31.94%) were mapped to the antisense strand from the four libraries. Moreover, 160,462,722, averaging 39.04%, were nonspliced reads, and 47,570,611 (avg. 14.54%) were mapped to spliced reads (Table 2). The mapping of clean reads to various genome regions in each sample mainly occurred in the exonic region followed by the intergenic and intronic (Supplemental Figure S3).

**TABLE 2:** Summary of sequencing results mapped to the mulberry reference genome

	Control samples		Drought stress samples	
	CK1	CK2	E1	E2
Total reads	81,866,870	81,783,646	81,016,592	82,478,570
Total mapped	56,053,795 (68.47%)	51,564,909 (63.05%)	54,614,925 (67.41%)	57,172,110 (69.32%)
Multiple mapped	3,015,259 (3.68%)	2,673,839 (3.27%)	2,664,837 (3.29%)	3,018,471 (3.66%)
Uniquely mapped	53,038,536 (64.79%)	48,891,070 (59.78%)	51,950,088 (64.12%)	54,153,639 (65.66%)
Read 1	26,608,166 (32.5%)	24,484,784 (29.94%)	26,092,993 (32.21%)	27,128,732 (32.89%)
Read 2	26,430,370 (32.28%)	24,406,286 (29.84%)	25,857,095 (31.92%)	27,024,907 (32.77%)
Reads map to (+)	26,403,373 (32.25%)	24,381,641 (29.81%)	25,814,658 (31.86%)	26,947,887 (32.67%)
Reads map to (−)	26,635,163 (32.53%)	24,509,429 (29.97%)	26,135,430 (32.26%)	27,205,752 (32.99%)
Nonsplice reads	41,311,279 (50.46%)	37,799,909 (46.22%)	39,393,358 (48.62%)	41,958,176 (50.87%)
Splice reads	11,727,257 (14.32%)	11,091,161 (13.56%)	12,556,730 (15.5%)	12,195,463 (14.79%)
Reads mapped in proper pairs	44,449,456 (54.29%)	40,938,322 (50.06%)	43,144,792 (53.25%)	45,117,968 (54.7%)
Proper‐paired reads map to different chromosome	0 (0%)	0 (0%)	0 (0%)	0 (0%)

*Note*. Reads map to (+) means the number of reads aligned to the positive strand (sense strand) on the genome; reads map to (−) means reads aligned to the negative strand (antisense strand) on the genome.

### 3.3 Identification of the mulberry Yu‐711 lncRNAs and mRNAs

**FIGURE 2:**
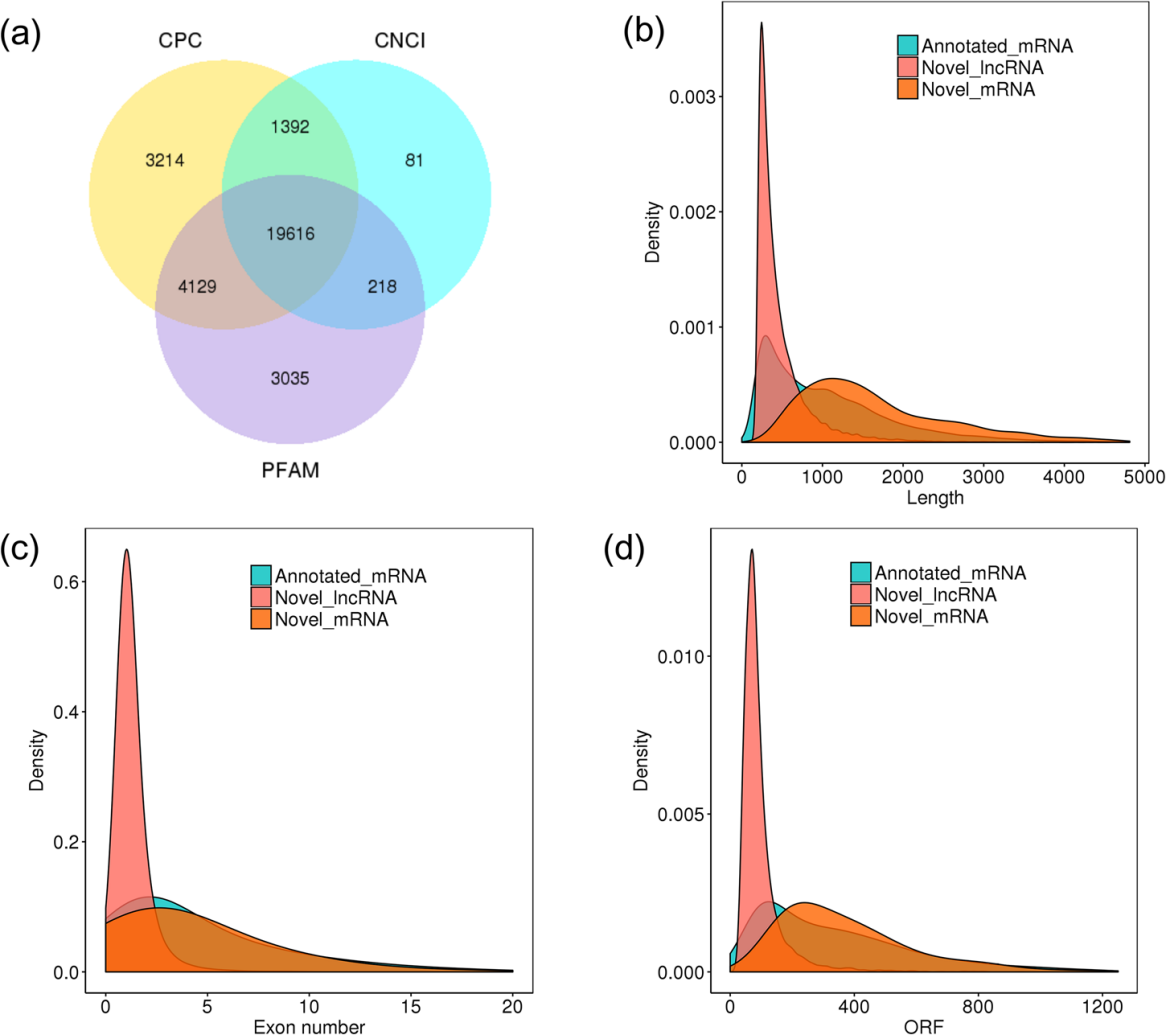
General characteristics of the long noncoding RNAs (lncRNAs) in *Morus alba*. (a) lncRNAs predicted by Coding Potential Calculator (CPC), Coding‐Noncoding Index (CNCI), and Pfam analysis; (b) lncRNAs length distribution compared with messenger RNAs (mRNAs); (c) lncRNAs exon number compared with mRNAs; (d) lncRNAs open reading frame (ORF) number compared with mRNAs

Transcripts with exon number >1, length >200 bp, and FPKM value >0.5 were chosen for additional analysis to identify probable lncRNAs among the uniquely mapped transcripts. The selected transcripts were evaluated for coding‐potential capacity by CPC, CNCI, and Pfam as described in the Long Noncoding RNA Identification and Classification section. Finally, we ended up with 19,616 novel lncRNAs (Figure 2a; Supplemental Table S1), 28,755 mRNA (including 26,965 annotated and 1,790 novel mRNA), and 12,022 unclassified (Supplemental Table S1). More lncRNAs were expressed than novel mRNAs but less than the annotated mRNAs under drought stress, and all the identified lncRNAs are novel.

### 3.4 Genomic characteristics and classification of the mulberry Yu‐711 lncRNAs and mRNAs

**FIGURE 3:**
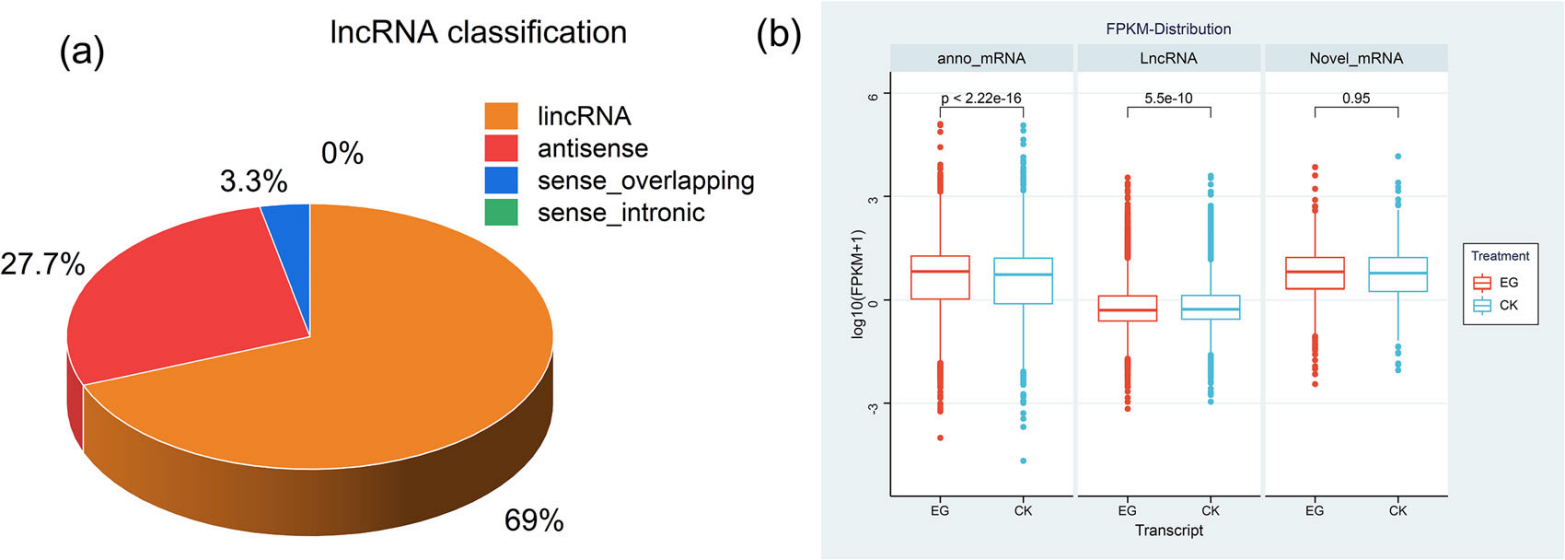
(a) Classification of long noncoding RNA (lncRNA) type distributed in the genome; (b) analysis of transcripts expression distribution in control and drought‐stress. EG, drought stress; CK, control

A comparison of the putative lncRNAs to the mRNAs was made by exploring their genomic characteristics. The results reveal that the novel lncRNA average length was ∼483 bp, shorter than the annotated mRNA with an average length of 1,128 bp and the novel mRNA with an average length of 1,761 bp (Figure 2b). Also, the novel lncRNAs exon number and ORF are shorter than the mRNAs. The lncRNAs had an average exon number of 1.2 compared with 4.2 and 4.6 of novel and annotated mRNA, respectively (Figure 2c). In addition, the ORF of lncRNA is concentrated within 400 amino acids compared with novel and annotated mRNA with a wide range of ORF lengths (Figure 2d). The putative lncRNAs obtained in the CK and EG groups were classified according to the HUGO Gene Nomenclature Committee (Wright, 2014) nomenclature guidelines for lncRNA, and the candidate novel_lncRNA were finally screened and named according to their location relationship with the coding gene; the novel_lncRNA of this analysis was obtained and classified into lincRNA (13,532; 69%), antisense (5,432; 27.7%), and sense overlapping (652; 3.3%) (Figure 3a).

The lncRNAs’ abundance in white mulberry leaves cells was lower than that of the annotated mRNAs but more than the novel mRNAs. Seven lncRNAs were randomly chosen for PCR assays to confirm the candidates lncRNAs from the sequencing data. The results indicated that all seven lncRNAs were successfully amplified with the expected band size in white mulberry leaves (Supplemental Figure S4). The transcripts expression obtained from the CK and EG groups were normalized to FPKM values. The expression pattern of the transcript between the EG and CK groups shows that lncRNA exhibits a lower expression level than the mRNAs, and there was a significant difference between the EG and CK groups (Figure 3b). In addition, the lncRNAs expression in the EG was significantly higher than in the CK. However, the annotated mRNAs expression in the EG is significantly lower than in the CK, whereas there is no significant difference in the novel mRNA expression between the EG and the CK groups (Figure 3b).

### 3.5 Differentially expressed lncRNAs and mRNAs under drought stress and control

**FIGURE 4:**
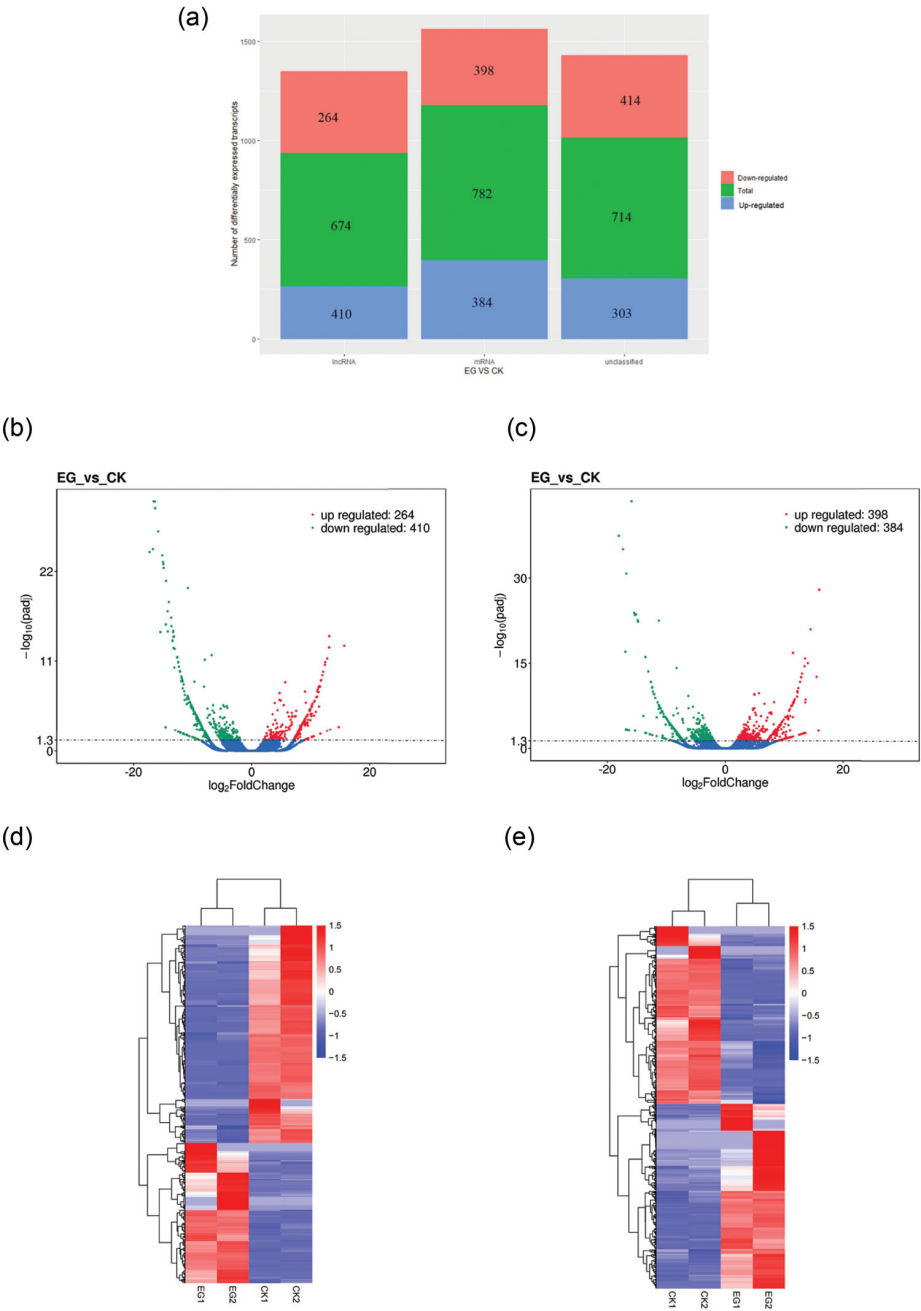
Differentially expressed long noncoding RNAs (DElncRNAs) and messenger RNAs (DEmRNAs) associated with *Morus alba* (Yu‐711) under drought stress and control. (a) Stack bar of differentially expressed transcripts; (b) Volcano plot of differentially expressed lncRNAs; (c) Volcano plot of differentially expressed mRNAs (d) Hierarchical clustering analysis of differentially expressed lncRNAs; (e) Hierarchical clustering analysis of differentially expressed mRNAs. The relative expression level of lncRNAs and mRNAs is represented in a low level (−1.5, blue) to a high level (1.5, red). The blue color indicates low concentration, and the red color means high expression concentration

To compare the expression levels of mRNAs or lncRNAs between the EG and CK leaves, we normalized the frequency of lncRNAs and mRNAs to FPKMs and analyzed the sequencing transcripts. Transcripts with adjacent *p* < .05 were identified as DEmRNAs and DElncRNAs. To visually show the relationship of these potential differential transcripts between EG and CK, a Venn diagram was used to reveal the situation between the two samples' comparisons (Supplemental Figure S5). We obtained 16,504 potential DElncRNAs between EG and CK and 1,147 and 1,960 were expressed only in EG and CK samples, respectively (Supplemental Figure S5a). In the mRNA, 20,203 potential differential mRNA was expressed between EG and CK and 972 and 1,194 in EG and CK, respectively (Supplemental Figure S5b). As a result, we obtained 674 DElncRNAs (264 upregulated and 410 downregulated) and 782 DEmRNAs (398 upregulated and 384 downregulated) (Figure 4a, Supplemental Table S2). The most affected upregulated DElncRNAs include LINC8729 and LINC11241, with log_2_ fold change >14.8 and 15.7, respectively, whereas TCONS_00019771 and TCONS_00019770 were the most affected downregulated, with log_2_ fold change less than −17.3 and −16.7, respectively.

Volcano plots were employed to show the relationships between false discovery rate and fold change of all the identified transcripts between the EG and CK plants (Figure 4b,c). In addition, hierarchical clustering heat map analyses (Figure 4d,e) indicate the systematic variation of mRNA or lncRNA expression between EG and CK. Principal component analysis was used to determine the distribution of the samples based on their expression characteristics. The principal component analysis charts demonstrated that samples from the same treatment were all grouped. For example, PC1 shows vast variation between EG and CK (61.70%) (Supplemental Figure S6).

### 3.6 Functional characteristics of DEmRNAs

**FIGURE 5:**
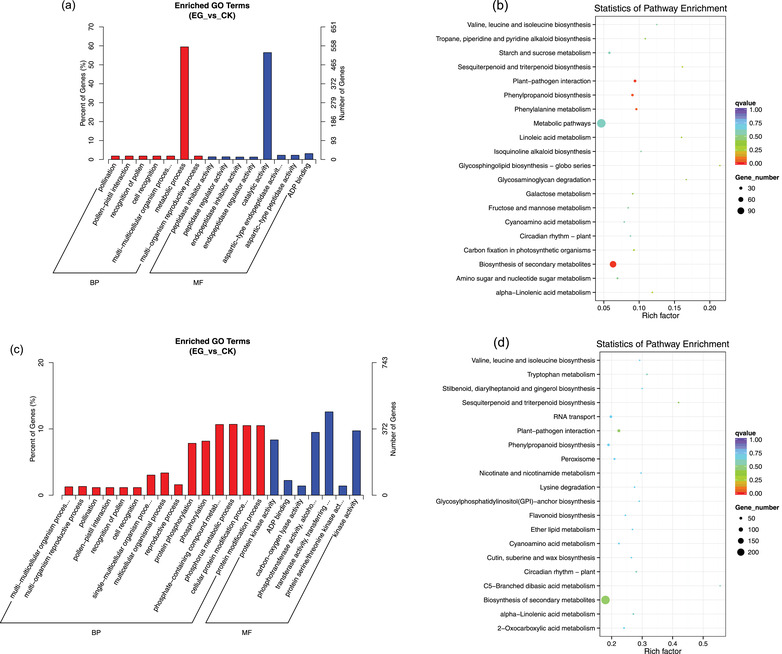
(a) Gene ontology (GO) function enrichment analysis and (b) KEGG pathway enrichment of differentially expressed messenger RNAs (DEmRNAs) identified in drought stress vs. control (EG_vs_CK) plants; (c) GO terms of messenger RNAs (mRNAs) collocated with differentially expressed long noncoding RNAs (DElncRNAs); (d) KEGG enrichment pathways of mRNAs collocated with DElncRNAs. BP, biological process; MF, molecular function. The ordinates in the figure represent different pathways, and the horizontal coordinates represent the proportion of genes represented by significant differences in the corresponding pathways to all genes in the pathway. The circle size represents the number of genes enriched in the corresponding pathway, and the larger the circle, the more genes enriched in that pathway. Color represents rich significance, and the closer to red, the more significant it becomes

The DEmRNAs were classified into two groups based on the GO classification annotations: biological process and molecular function. The DEmRNAs with the metabolic process (GO:0008152), multiorganism reproductive process (GO:0044703), and cell recognition (GO:0008037) were among the significantly enriched GO terms in the biological process between EG and CK (Figure 5a; Supplemental Table S3). The DEmRNAs related to the catalytic activity (GO:0003824), endopeptidase inhibitor activity (GO:0004866), peptidase inhibitor activity (GO:0030414), and ADP binding (GO:0043531) were significantly enriched in the molecular function (Figure 5a). The catalytic activity terms contain a lot of DEmRNAs, suggesting that the drought stress may have regulated genes involved in enzymatic activities.

The KEGG analysis for the mRNAs produced by EG and CK discovered 603 DEmRNAs connected with KEGG pathways. The top 20 ranked KEGG pathways are shown in Figure 5b. The most significant enrichment among the top 20 KEGG pathways includes biosynthesis of secondary metabolites (pop01110), plant–pathogen interaction (pop04626), phenylpropanoid biosynthesis (pop00940), and others (Zhang, Liang et al., 2020; Ackah et al., 2021) (Figure 5b; Supplemental Table S3). The gene (*L484_020672*) encoding CSY2 protein, a crucial stress response protein in plants, was upregulated by 13.4‐fold, suggesting the possible activation of glyoxylate cycle and citric acid cycle pathways under the drought stress. On the other hand, the gene (*L484_013081*) encoding PER47 protein, an essential protein against stresses such as oxidative stress (H_2_O_2_), was upregulated by 9.2‐fold. Moreover, the gene encoding DFR protein (*L484_000974*) was downregulated by 8.9‐fold, suggesting that the flavonoid metabolism pathway might have been suppressed during the drought stress. Genes encoding PER45 (*L484_002294*) and PER43 (*L484_024144*) were downregulated by 7.2‐fold. These protein genes are crucial in combatting oxidative stress.

### 3.7 Biological functional analyses of the DElncRNA–target genes

Studies have shown that at the *cis‐*acting loci, lncRNAs can inhibit or activate the expression of genes close to the lncRNAs’ transcription sites on the same chromosome (Zhang, Liang et al., 2020). Colocation means that lncRNA may have a regulatory effect on adjacent protein‐coding genes and is analyzed by looking for genes within 100 kb upstream and downstream of lncRNAs. Collocation analysis is performed to predicted function role of lncRNAs target genes if the experimental sample size is less than or equal to five Therefore, to further understand the function model of *cis‐*acting lncRNAs in our study, we looked for mRNAs located 100 kb upstream and 100 kb downstream of DElncRNAs transcription sites. We examined the sequences of 674 DElncRNAs for their *cis*‐regulatory effects and classified the interaction between the lncRNA and its surrounding mRNA as collocated. The result indicated that a total of 6,125 mRNAs were collocated with the 674 DElncRNAs, implying that DElncRNAs may influence the expression of these 6,125 mRNAs via their *cis*‐regulatory functions (Supplemental Table S4). Remarkably, one DElncRNA could collocate several mRNAs and vice versa. For instance, DElncRNA (TCONS_00087200, TCONS_00077793, and TCONS_00073297) were collocated to 36, 34, and 31 mRNAs, respectively. Also, genes, including *L484_021836* (encode for KH domain‐containing protein) and *L484_021844* (encode for Receptor‐like protein 12), collocate with many DElncRNAs (Supplemental Table S4). These imply the complex gene regulatory network at the *cis*‐regulatory loci.

The GO classification of these DElncRNAs’ putative target genes revealed their involvement in various processes including biological processes and molecular function (Figure 5c). In total, 2,207 target genes were mapped to the KEGG database, and the top 20 enriched KEGG pathways were analyzed (Figure 5d; Supplemental Table S4). These findings reveal that the collocated mRNAs involved in the biosynthesis of secondary metabolites, sesquiterpenoid and triterpenoid biosynthesis (pop00909), and plant–pathogen interaction were significantly enriched in the pathways (Nam et al., 2019). Studies have shown that the biosynthesis of secondary metabolites is one of the mechanisms plants use to counteract environmental stresses (Isah, 2019). In this study, we noticed 245 target genes in the biosynthesis of secondary metabolites (pop01110) and 13 in the sesquiterpenoid and triterpenoid biosynthesis (pop00909) were collocated with the DElncRNA (Figure 5d; Supplemental Table S4), suggesting that drought stress response in mulberry can result in regulation of several genes related to the biosynthesis of secondary metabolites pathways.

Furthermore, we searched for the DElncRNAs collocated with DEmRNAs using the most enriched pathways in the top 20 including biosynthesis of secondary metabolites and sesquiterpenoid and triterpenoid pathways. Interestingly, 30 DEmRNAs targets were identified. However, these 30 DEmRNA targets were collocated with 56 DElncRNAs to form the DElncRNA–DEmRNA target pairs (Supplemental Table S9), and therefore we hypothesize that these 56 DElncRNAs may regulate these 30 DEmRNAs. Furthermore, a gene (*L484_009248*) that encodes for ATP‐citrate synthase beta chain protein 1 was collocated with TCONS_00081189, which are involved in the biosynthesis of secondary metabolites pathways. The ATP‐citrate synthase beta chain protein is a crucial functional protein and has been reported to play an active role in plant response to environmental stresses (Batista et al., 2017). This finding may indicate that TCONS_00081189 may regulate the host secondary metabolites pathway in providing carbon and energy through the *cis*‐regulation of L484_009248 by 5.10‐fold. A gene, *L484_024421*, encoding peroxidase 21, was significantly collocated with TCONS_00073297 and upregulated by 2.81‐fold. The target DEmRNAs were generally involved in the up‐ and downregulation patterns (Supplemental Table S9).

### 3.8 Analysis of DElncRNA–mRNA target genes predicted network

**FIGURE 6:**
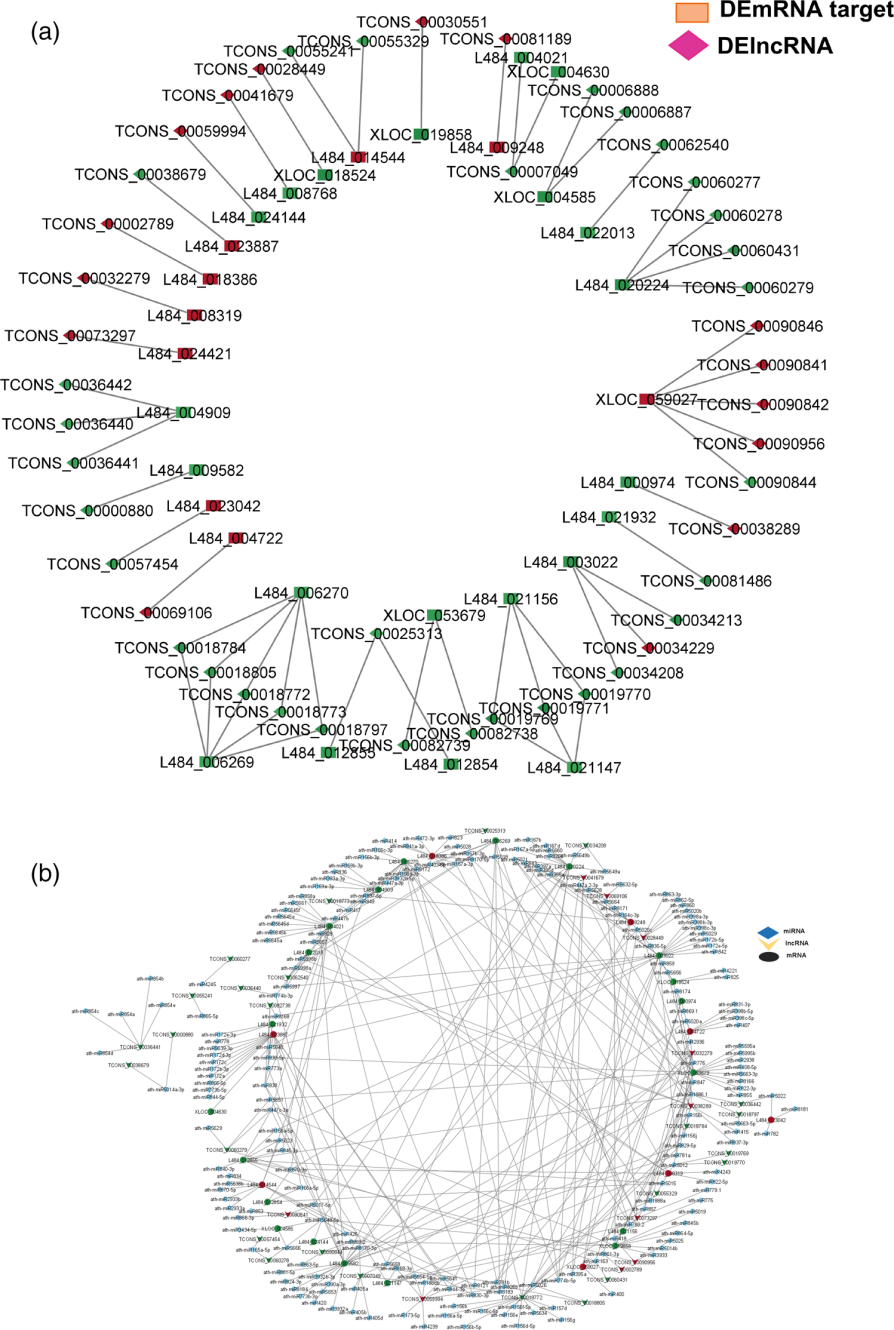
Display of the differentially expressed long noncoding RNAs (DElncRNAs)–messenger RNAs (mRNAs) targets regulatory networks in response to drought stress. (a) DElncRNAs–differentially expressed messenger RNAs (DEmRNAs) target regulatory networks from the biosynthesis of secondary metabolites pathway. Rectangle shape indicates DEmRNAs, and the diamond shape indicates DElncRNAs, red indicates upregulation, and green indicates downregulation. (b) Display of the predicted DElncRNAs–miRNAs–DEmRNAs regulatory networks. V‐shapes indicate lncRNAs, diamonds indicate miRNAs, and eclipse indicates DEmRNAs. Red color indicates upregulation and green color indicates downregulation

Long noncoding RNA regulates its target gene at the *cis‐* and *trans*‐regulatory loci. To understand the DElncRNAs network on the target mRNA, we constructed a gene network involving the DElncRNA–mRNA target and DElncRNA–DEmRNA target using the target genes involved in the biosynthesis of secondary metabolites pathways. The biosynthesis of secondary metabolites pathways was the most enriched and contained many target genes in the KEGG pathway and hence was selected to perform further analysis on the gene network. From the results, we found that 228 DElncRNA formed a gene network with 237 mRNA targets (Supplemental Table S5). The DElncRNA–DEmRNA target network analysis formed 76 nodes with 56 edges. In addition, 15 and 31 DElncRNAs were up‐ and downregulated, respectively, whereas nine DEmRNAs were upregulated and 21 were downregulated (Figure 6a; Supplemental Table S9).

### 3.9 Analysis of DElncRNA and DEmRNA target as miRNA precursors

The lncRNA and mRNA loci overlapped with miRNA loci on the same strand are considered the miRNA precursors (Wilusz et al., 2009; Dai et al., 2018). Therefore, we compared the DElncRNA–DEmRNA target sequences with the miRNA sequences obtained from miRBase to determine whether lncRNAs or mRNA are, in fact, precursors of miRNAs. The results show that 36 DElncRNAs recruited 96 miRNA precursors (Supplemental Table S6); one DElncRNA could recruit many miRNAs. For instance, TCONS_00069106 overlapped with ath‐miR447a.2‐3p, ath‐miR5628, and ath‐miR5632‐5p. On the contrary, one miRNA could be recruited by several lncRNAs; for example, ath‐miR1886.1 overlapped with TCONS_00028449 and TCONS_00038289, and ath‐miR447a.2‐3p could recruit TCONS_00038289 and TCONS_00069106. On the other hand, the 30 DEmRNA targets recruited 258 miRNAs (Supplemental Table S6). Gene regulatory networks involving DElncRNA–miRNA and DEmRNA–target miRNA were constructed and visualized in Cytoscape 3.9.1 (Supplemental Figure S7). Interestingly, 63 DElncRNA–DEmRNA formed a network with 215 miRNAs to form 278 nodes of the DElncRNA–miRNA–DEmRNA network with a total of 370 network edges (Figure 6b).

### 3.10 Validation of DElncRNA and DEmRNA–targets genes in response to drought stress

**FIGURE 7:**
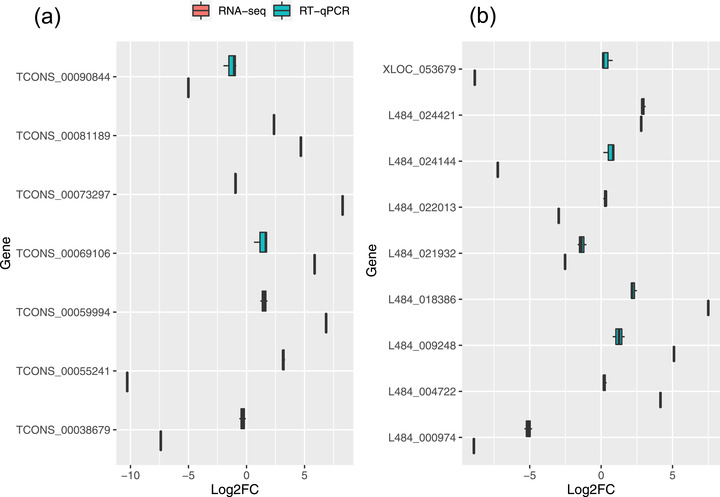
Quantitative real‐time polymerase chain reaction (qRT‐PCR) for the relative expression level and RNA sequencing (RNA‐seq) results of (a) differentially expressed long noncoding RNAs (DElncRNAs) and (b) differentially expressed messenger RNAs (DEmRNAs) target genes. The mulberry actin3 (*HQ163775.1*) gene was used as the internal control gene. All the samples were conducted with three independent biological replicates and three technical replicates. The relative expression levels were calculated as 2^−∆∆CT^, where ∆∆C_T_ = ΔCT(a target sample) − ΔCT(a reference sample). The *y* axis represents the lncRNAs or mRNAs, and the *x* axis denotes the log_2_ foldchange of relative expression or fragments per kilobase of transcript per million mapped reads

To validate the Illumina sequencing data of the mulberry transcriptome and the expression patterns of the DEmRNAs and DElncRNAs, nine DEmRNAs target genes and seven DElncRNAs were selected for qRT‐PCR analyses. The criteria for the DEmRNAs and the DElncRNAs were based on the differentially expressed genes that actively participated in the biosynthesis of secondary metabolites pathways as mentioned above. The mulberry *actin3* gene was used as an internal reference for the relative quantification as its expression level changes upon drought stress treatment. The seven randomly selected DElncRNAs included four upregulated (TCONS_00081189, TCONS_00069106, TCONS_00073297, and TCONS_00059994) and three downregulated (TCONS_00038679, TCONS_00055241, and TCONS_00090844) (Figure 7a). Furthermore, the nine DEmRNAs, including four upregulated (L484_009248, L484_004722, L484_024421, and L484_018386) and five downregulated (L484_024144, L484_000974, L484_022013, L484_021932, and XLOC_053679) (Figure 7b) were also validated by qRT‐PCR. Altogether, these data suggest that the transcriptome results are reliable and can represent the virtual expression pattern upon drought stress treatment. The phylogenetic relationship of the transcript sequence is shown in Supplemental Figure S8.

### 3.11 Analysis of alternative splicing events stress

Alternative splicing is important in plants’ resistance to abiotic stressors including drought (Song et al., 2020). Some precursor mRNAs have multiple shearing procedures to form different mRNAs, an important mechanism for regulating gene expression and generating protein diversity. We used the RNA‐seq data to analyze the five types of AS events in plants using rMATS software (http://rnaseq‐mats.sourceforge.net/index.html) (Shen et al., 2014). The five types of AS analyzed include intron retention, alternative 3′ splicing site, skipped exon (SE), alternative 5′ splicing site, and mutually exclusive exon (MXE). Interestingly, only two types of AS events, SE and MXE, occurred in the mulberry plant's response to drought stress. Among the two AS events, SE was the major type (Supplemental Table S7). Furthermore, 113 SE‐type and eight MXE‐type AS events were identified under drought stress (Supplemental Figure S9). The results indicate that the SE‐type of AS event may be more involved in mulberry plant response to drought stress.

### 3.12 Protein–protein interaction analysis

Proteins in plant cells and tissue do not function as individual molecules but rather in the context of networks where they play coordinated and interconnected roles (Zenda et al., 2018). Therefore, we investigated several differential gene products using the online String 10.5 database to ascertain how mulberry leaf drought stress signals are relayed via protein–protein interactions to alter certain cellular functions (Supplemental Figure S10).

## 4 DISCUSSION

Multiple abiotic stresses, including drought, cold, salinity, and many others, inhibit plant growth and development. Therefore, to adapt to changes in the environment, plants must optimize their development and metabolism (Leng et al., 2020). Tolerance to stress in plants is a complex physiological feature governed by many genes. Therefore, studying a plant's physiological reactions to stress is critical for agricultural development and production practices. Because of various modern molecular breeding techniques, increasing crop tolerance to environmental cues has been possible. For instance, the advancements in next‐generation sequencing technologies have enabled the identification of lncRNAs in various plant species on a genome‐wide level (Nejat and Mantri, 2018; Leng et al., 2020). Numerous studies have recently revealed that lncRNAs play critical roles in various fundamental biological processes including the regulation of mRNA processing and transcription mechanisms such as splicing, editing, localization, translation, and turnover (Geisler and Coller, 2013; Kashi et al., 2016). In addition, lncRNAs play important biological processes such as transcriptional gene silencing (Wierzbicki, 2012), protein‐coding gene expression regulation (Zhang, Liao et al., 2014), and regulation of epigenetic and chromatin structure in plants (Magistri et al., 2012).

This study used high‐throughput sequencing technology to analyze the lncRNA of mulberry (Yu‐711) leaves under drought stress and control. In total, 19,616 novel lncRNAs were identified. Among them, 264 were upregulated and 410 were downregulated. Characteristically, the mulberry (Yu‐711) lncRNAs shared some features with other species, for instance, relatively low expression levels and low exon numbers (Zhang, Liao et al., 2014). Furthermore, the mulberry lncRNA sequences are shorter than the mRNAs (Wang et al., 2018). The average length of the lncRNAs was 483 bp compared with the average length of the protein‐coding genes (1,128 bp).

Depending on the location of lncRNAs to the protein‐coding genes loci in the genome, they are mostly classified into various classes including lincRNAs (transcribed from within the gene's intergenic region), antisense (across from the protein‐coding gene's antisense strand), and intronic (derived from within the protein‐coding gene's intronic region) (Jin et al., 2020, Rai et al., 2019). Our results show that the lincRNAs class was the most‐expressed lncRNA of the total lncRNAs in the mulberry leaves during drought stress (Figure 2e) (Zhang, Yin et al., 2020). More expression level of lincRNAs suggests that mulberry uses lincRNAs to regulate the target mRNAs under drought stress. The lncRNAs can contain either DNA‐ or protein‐binding sites or both and can be involved in various biological processes (Zhang, Yin et al., 2020). They can exert regulatory functions via *cis*‐ or *trans*‐regulation (Deng & Meller, 2006; Zhang, Liang et al., 2020). Our collocated analysis reveals that 6,125 target mRNAs were collocated with the 674 DElncRNAs.

The biologically regulatory functions of the target mRNAs analysis via GO and KEGG analysis reveal that the potential target genes were implicated in biological processes and molecular function. In the biological process, the target genes include protein modification process (GO:0036211), cellular protein modification process (GO:0006464), phosphorus metabolic process (GO:0006793), multi‐multicellular organism process, and many others. In the molecular function, the target mRNAs were mainly involved in transferase activity (GO:0016740), kinase activity (GO:0016301), protein kinase activity (GO:0004672), phosphotransferase activity, ADP binding (GO:0043531), and others. In addition, we discovered that a single gene could participate in dozens of different pathways. For instance, *L484_009248*, *L484_024421*, and *L484_022013* were enriched with various functional elements involved in molecular and biological processes. Moreover, on the other hand, multiple candidate genes were enriched for the same functional category. For instance, the candidate genes *L484_009248*, *L484 _024421*, and *L484_ 022013* had the same functional terms, implying that these genes and their associated GO terms may require for the mulberry plant response to drought stress.

The KEGG pathway analysis revealed that DElncRNAs regulate 117 pathways of the target genes (Supplemental Table S4). However, the top 20 KEGG pathway enrichment analysis shows that the target genes were significantly enriched in the biosynthesis of secondary metabolites (245 target genes), sesquiterpenoid and triterpenoid biosynthesis (13 target genes), plant–pathogen interaction (57 target genes), tryptophan metabolism (12 target genes), circadian rhythm–plant (16 target genes), and other pathways (Figure 5). Because of the substantial number of target genes involved in the biosynthesis of secondary metabolites in the KEGG pathways, we further analyzed the target genes involved in this pathway. We found that most target genes in this pathway were differentially expressed genes (DEGs); however, most of these genes were also involved in the other pathways. The biosynthesis of secondary metabolites is one of the mechanisms plants use to counteract environmental stresses (Isah, 2019). Differentially expressed genes such as *L484_018386* (encode beta‐glucosidase 12), *L484_009248* (encode ATP‐citrate synthase beta chain protein 1), *L484_004722* (encode LL‐diaminopimelate aminotransferase), and *L484_024421* (encode peroxidase 21) in this pathway were all upregulated during the drought stress indicating secondary metabolites may have played a key role in the plant response to the drought stress condition. These four up‐regulated genes, L484_018386, L484_009248, L484_004722, and L484_024421, were found to be collocated at 11.9 kbp upstream of TCONS_00002789 (upregulation), 23.9 kbp upstream of TCONS_00081189 (upregulated), 333 bp upstream of TCONS_00069106 (upregulated), and 39.4 kbp downstream of TCONS_00073297 (upregulated), respectively. Also, downregulated target DEGs including L484_021932 (encode jasmonate O‐methyltransferase), L484_000974 (encode bifunctional dihydroflavonol 4‐reductase/flavanone 4‐reductase), L484_022013 (encode dehydrodolichyl diphosphate synthase 2), L484_024144 (encode peroxidase 43), and XLOC_053679 were also found collocated at 51.2 kbp downstream of TCONS_00081486 (downregulated), 25.92 kbp downstream of TCONS_00038289 (upregulated), 56.24 kbp upstream of TCONS_00062540 (downregulated), 62.20 kbp downstream of TCONS_00059994 (upregulated), and 5.91 kbp downstream of TCONS_00082738 (downregulated), respectively. Thus, these results suggest that the mulberry plant under the drought stress response may use the lncRNAs‐mediated strategy. However, additional investigations are required to confirm the network between these DElncRNAs and their target DEGs.

MicroRNAs are small RNAs that act as transcriptional and posttranscriptional regulators of messenger RNAs. Several studies demonstrate that lncRNA contains sequences similar to their target miRNA and acts as an indirect regulator of target mRNA miRNA expression by competing for shared miRNA (Parker et al., 2011; Rubio‐Somoza et al., 2011). For example, in wheat plants, lncRNAs TalnRNA5, TapmlnRNA8, and TapmlnRNA19 have been reported to act as miRNA precursors (Nejat and Mantri, 2018). In this study, we predicted whether the DElncRNAs or DEmRNAs target transcripts might act as miRNA precursors considering the DElncRNAs and the DEmRNAs target involved in the biosynthesis of the secondary metabolite pathway (Supplemental Figure S6). For instance, ath‐miR447a.2‐3p was predicted to interact with TCONS_00069106, TCONS_00038289, and TCONS_00018772. These lncRNAs were also linked to mRNA targets that code for LL‐diaminopimelate aminotransferase, bifunctional dihydroflavonol 4‐reductase/flavanone 4‐reductase, and hypothetical protein L484_006270, respectively. miR447a has been reported to differentially express and regulate its target genes during leave senescing (Thatcher et al., 2015). In Sichuan‐pepper (*Zanthoxylum bungeanum* Maxim.) response to drought, miR447a‐3p regulates its target gene L‐ascorbate peroxidase3 (Gelaw & Sanan‐Mishra, 2021). Also, miRNAs such as miR1888a & b and miR1886 were predicted to target several DElncRNAs. The predicted miRNAs were mainly involved in cleavage, and few were involved in translation. A recent report reveals that miRNAs and their target genes are involved in secondary metabolites pathways in response to drought stress (Gelaw & Sanan‐Mishra, 2021).

The construction of lncRNAs–miRNAs–mRNAs reveals the complex gene regulatory network involved in the mulberry plant's response to drought stress (Figure 6). We speculate that the mulberry plant could use the lncRNA–miRNA–mRNA regulatory network mechanism to promote response to drought stress by producing secondary metabolism. However, the regulatory role of the miRNAs needs to be examined experimentally. The interaction of lncRNAs and their target mRNAs was verified by the LncTar database. LncTar (http://www.cuilab.cn/lnctar) is a fast and accurate method for predicting lncRNA targets (Li et al., 2015). LncTar's primary advantage is that it can accurately predict the interaction of lncRNAs with target genes, which is crucial for understanding the function and molecular mechanisms of lncRNAs (Leng et al., 2020). We predicted that six lncRNAs interact with six candidate genes using a threshold of −0.05 (Supplemental Table S8).

Interestingly, we found that only L484_009248–TCONS_00081189 has a threshold greater than −0.1 (Supplemental Table S8) in our results, implying that there may exist a strong correlation between the lncRNAs and candidate genes. We further validated the lncRNAs and candidate genes by qRT‐PCR analysis. The results reveal that most of the target genes, including those that interacted with the lncRNAs, were expressed in the mulberry leaves under drought stress and further confirm the RNA‐seq data's reliability (Figure 6a,b). Phylogenetic analysis of the lncRNAs and candidate genes sequence reveals that the target gene *L484_022013*, which encodes dehydrodolichyl diphosphate synthase 2 protein, is closely related to a novel target gene (*XLOC_053679*). Dehydrodolichyl diphosphate synthase is a key enzyme involved in the biosynthesis of dolichols, which play an important cellular function in plants against environmental stress (Cunillera et al., 2000; Zhang et al., 2008). Also, *L484_009248* (ATP‐citrate synthase beta chain protein 1) and *L484_021932* (Jasmonate O‐methyltransferase protein) were close related (Supplemental Figure S7). ATP citrate lyase produces cytosolic acetyl‐CoA precursors for thousands of specialized plant metabolites, including waxes, sterols, and polyketides (Li‐Beisson et al., 2013). ATP citrate expression in plants under drought stress has been reported to increase (Prinsi et al., 2018), which is consistent with our results.

Alternative splicing has been implicated in plants’ tolerance to abiotic stresses, including drought (Song et al., 2020). Multiple shearing processes are performed on the same precursor mRNA to generate distinct mRNAs, a key method for controlling gene expression and producing protein variety. Our results reveal that about 14.54% of the transcriptome data underwent AS events. In plants, all the five types of AS events, including intron retention, alternative 3′splicing site, skipped exon (SE), alternative 5′ splicing site, and mutually exclusive exon (MXE), occur (Song et al., 2020). However, we discovered only SE and MXE. The genes involved were mostly protein kinases, including the receptor interacting protein kinases and calcineurin B‐like protein (CBL) and CBL interacting protein kinases (CIPKs) (Supplemental Table S7). The CIPKs are protein kinases and facilitate signal transduction primarily through the phosphorylation of proteins. Extensive research in *Arabidopsis* has established the role of calcineurin B‐like protein–CIPK complexes in response to abiotic and biotic stressors and nutritional deprivation (Yu et al., 2014; Sanyal et al., 2016). Receptor interacting protein kinases are a family of Ser/Thr/Tyr kinases whose functions are implicated in gene regulation (Cuny and Degterev, 2021). Altogether, these results imply that mulberry genes may undergo AS events under drought stress to produce protein kinases to combat the drought stress effect; however, further study is required.

Proteins are typically present in complexes within the cell, and interactions between diverse proteins regulate biological activities (Zenda et al., 2018). The predicted protein interaction of the drought response genes identified has strong interaction (Supplemental Figure S10). Proteins such as phosphoenolpyruvate carboxykinase (ATP) (L484_025529), glucose‐6‐phosphate isomerase (L484_021184), and tyrosine aminotransferase (L484_010311) forms the central role of the protein–protein network and interacts with other proteins. These proteins are significantly involved in the biosynthesis of secondary metabolites pathways and other pathways such as carbon fixation in photosynthetic organisms and play a crucial role in drought stress response.

## 5 CONCLUSION

This study detected 674 DElncRNAs and 782 DEmRNAs in mulberry leaves in response to drought stress using deep RNA‐seq. Functional investigation of DElncRNA target genes and the DElncRNA–DEmRNA regulation network revealed that the DElncRNAs might participate in the biosynthesis of secondary metabolites under mulberry response to drought stress. In addition, we validated seven DElncRNAs by PCR with the desired band size. Seven DElncRNAs and nine target DEGs were confirmed to respond to drought stress by qRT‐PCR and were involved in the biosynthesis of secondary metabolites and other pathways. Also, six lncRNAs interacted with six target genes as verified by LncTar. Our network data may provide resources for studying the roles of lncRNAs associated with mulberry and may shed light on the mechanisms behind the mulberry*–*abiotic stress interactions. Additional research is necessary to elucidate the drought tolerance mechanism of candidate genes and lncRNAs. Despite the limitations, our study adds new information regarding drought stress to aid in developing drought tolerance in mulberry.

## AUTHOR CONTRIBUTIONS

Michael Ackah: Conceptualization; Data curation; Formal analysis; Investigation; Methodology; Visualization; Writing–original draft; Writing–review & editing. Xin Jin: Resources; Visualization. Qiaonan Zhang: Resources; Validation. Frank Kwarteng Amoako: Data curation; Formal analysis; Software. Lei Wang: Data curation; Visualization. Thomas Attaribo: Data curation; Formal analysis; Visualization. Mengdi Zhao: Data curation; Resources. Feng Yuan: Visualization. Richard Ansah Herman: Data curation; Visualization. Changyu Qiu: Validation. Qiang Lin: Validation. Zhi Yin: Validation. Weiguo Zhao: Conceptualization; Funding acquisition; Project administration; Resources; Supervision; Writing–review & editing.

## CONFLICT OF INTEREST

The authors declare no conflicts of interest.

## Supporting information

Supplemental Figure S1. Pearson correlation between EG and CK samples. EG1‐EG2; drought stress treatment. CK1‐CK2; control treatment.
Supplemental Figure S2. Distribution of base content along with reads. Type A, T, G, and C are the individual nucleotide bases, and N is read containing unknown bases
Supplemental Figure S3. Reads distribution in different regions of the genome.
Supplemental Figure S4. Validation of candidate lncRNAs. 1 μg total RNA extracted from mulberry (Yu‐711) leaves was reverse transcribed into cDNA. Specific primers for seven lncRNAs were used to perform PCR. The PCR products were separated on 1% agarose gel to detect the expression of lncRNAs. Ck. control; a. TCONS_00081189; b. TCONS_00059994; c. TCONS_00038679; d. TCONS_00055241; e. TCONS_00073297; f. TCONS_00090844; g. TCONS_00069106
Supplemental Figure S5. Venn diagrams for two groups comparison of the differential transcripts expression patterns. (a) lncRNAs comparison in CK and EG. (b) mRNAs comparison in CK and EG. (c) unclassified transcripts comparison in CK and EG.
Supplemental Figure S6. Principal component analysis (PCA) of the samples.
Supplemental Figure S7. Predicted DElncRNAs‐miRNAs and DEmRNAs target‐miRNAs regulatory networks in response to drought stress. (a) DElncRNAs‐miRNAs regulatory networks from the biosynthesis of secondary metabolites pathway. The Triangle shape indicates miRNAs, and the eclipse shape indicates DElncRNAs, red indicates up‐regulation, and green indicates down‐regulation. (b) DElmRNAs target‐miRNAs regulatory networks. The Triangle shape indicates miRNAs, and the diamonds indicate DEmRNAs. Red color indicates up‐regulation, and green color indicates down‐regulation.
Supplemental Figure S8. Phylogenetic heatmap of the transcripts validated by quantitative real‐time PCR for the relative expression level of DEmRNA‐target genes (a) and DElncRNA (b). The trees were constructed with MEGA 11 software using the Neighbour‐Joining (NJ) method and Boostrap with 1000 replicates. TBtools software was used to build the heatmap.
Supplemental Figure S9. Distribution of AS event in mulberry plant response to drought stress and control.
Supplemental Figure S10. protein‐protein interaction network in response to drought stress in mulberry leaves. The lines represent the edges, and the V‐shape is the node

Supplemental Table S1: Identified transcripts (EG_CK) (an Excel workbook)

Supplemental Table S2: Differentially expressed transcripts and (an Excel workbook)

Supplemental Table S3: GO and KEGG enrichments of the Differentially expressed transcripts (an Excel workbook)

Supplemental Table S4: Target genes and KEGG pathways (an Excel workbook)

Supplemental Table S5: Differentially expressed target genes and lncRNAs pairs (an Excel workbook)

Supplemental Table S6: Differentially expressed transcripts and miRNAs pairs (an Excel workbook)

Supplemental Table S7: Alternative splicing events (an Excel workbook)

Supplemental Table S8: LncTar prediction and qRT‐PCR primers (an Excel workbook)

Supplemental Table S9: The co‐located DElncRNAs–DEmRNAs pairs (A word file)

## Data Availability

The original data sets used in this study are included in the article Supplemental materials; the raw sequence data have been deposited at the NCBI Sequence Read Archive (SRA) (https://ncbi.nlm.nih.gov/subs/sra; PRJNA808829). Other reasonable requests can be made to the corresponding author.
