# Supplementary material for: Long noncoding RNA transcriptome analysis reveals novel lncRNAs in Morus alba ‘Yu‐711’ response to drought stress
Source: Plant Genome. 2022 Oct 26;17(1):e20273. doi: 10.1002/tpg2.20273 (PMC12807294; doi:10.1002/tpg2.20273)
Supplement: Supplementary file 1 [file TPG2-17-e20273-s001.docx]

Table S9 The co-located DElncRNA–DEmRNA pairs

| **LncRNA_ID** | **lncRNA locus** | **regulation** | **log2 (FC)** | **mRNA_ID** | **mRNA locus** | **regulation** | **log2 (FC)** | **Gene product name** |
| --- | --- | --- | --- | --- | --- | --- | --- | --- |
| TCONS_00081189 | 42562-42824 | up | 4.69 | L484_009248 | 66703-73314 | up | 5.10 | ATP-citrate synthase beta chain protein 1 |
| TCONS_00060431 | 243280-264377 | down | -15.03 | L484_020224 | 303317-307449 | down | -13.11 | Acidic endochitinase |
| TCONS_00060279 | 309474-310225 | down | -7.39 | L484_020224 | 303317-307449 | down | -13.11 | Acidic endochitinase |
| TCONS_00060277 | 307729-308362 | down | -7.18 | L484_020224 | 303317-307449 | down | -13.11 | Acidic endochitinase |
| TCONS_00060278 | 308554-309102 | down | -7.16 | L484_020224 | 303317-307449 | down | -13.11 | Acidic endochitinase |
| TCONS_00069106 | 55305-56869 | up | 5.87 | L484_004722 | 50135-54971 | up | 4.16 | LL-diaminopimelate aminotransferase |
| TCONS_00073297 | 834821-835770 | up | 8.28 | L484_024421 | 792321-795440 | up | 2.81 | Peroxidase 21 |
| TCONS_00038679 | 698877-699293 | down | -7.39 | L484_023887 | 748077-757796 | up | 11.50 | Protein WAX2 |
| TCONS_00055241 | 75679-75973 | down | -10.28 | L484_014544 | 88469-93503 | up | 2.47 | Putative primary amine oxidase 2 |
| TCONS_00055329 | 154940-155166 | down | -7.26 | L484_014544 | 88469-93503 | up | 2.47 | Putative primary amine oxidase 2 |
| TCONS_00006888 | 446684-452290 | down | -11.02 | XLOC_004585 | 437695-452997 | down | -15.49 | - |
| TCONS_00006887 | 437749-443763 | down | -10.98 | XLOC_004585 | 437695-452997 | down | -15.49 | - |
| TCONS_00090844 | 262596-353218 | down | -5.01 | XLOC_059027 | 177740-205061 | up | 5.72 | - |
| TCONS_00090846 | 262691-352086 | up | 3.33 | XLOC_059027 | 177740-205061 | up | 5.72 | - |
| TCONS_00090841 | 255907-256401 | up | 5.35 | XLOC_059027 | 177740-205061 | up | 5.72 | - |
| TCONS_00090842 | 259508-311683 | up | 2.61 | XLOC_059027 | 177740-205061 | up | 5.72 | - |
| TCONS_00090956 | 259446-311852 | up | 2.19 | XLOC_059027 | 177740-205061 | up | 5.72 | - |
| TCONS_00032279 | 231540-233193 | up | 5.08 | L484_008319 | 228338-230495 | up | 5.13 | hypothetical protein L484_008319 |
| TCONS_00059994 | 250810-253744 | up | 6.86 | L484_024144 | 185870-188606 | down | -7.24 | Peroxidase 43 |
| TCONS_00028449 | 64622-66847 | up | 4.00 | XLOC_018524 | 12644-106852 | down | -16.83 | - |
| TCONS_00030551 | 164288-201475 | up | 12.51 | XLOC_019858 | 166326-267009 | down | -17.39 | - |
| TCONS_00062540 | 176982-177486 | down | -8.01 | L484_022013 | 233731-235482 | down | -2.97 | Dehydrodolichyl diphosphate synthase 2 |
| TCONS_00038289 | 27376-27986 | up | 3.86 | L484_000974 | 239-1458 | down | -8.91 | Bifunctional dihydroflavonol 4-reductase/flavanone 4-reductase |
| TCONS_00082738 | 95713-96430 | down | -8.81 | XLOC_053679 | 87448-89802 | down | -8.85 | - |
| TCONS_00082739 | 96939-97400 | down | -7.47 | XLOC_053679 | 87448-89802 | down | -8.85 | - |
| TCONS_00057454 | 825781-826125 | down | -7.18 | L484_023042 | 782975-788245 | up | 8.32 | (R)-mandelonitrile lyase 1 |
| TCONS_00002789 | 82243-87471 | up | 8.92 | L484_018386 | 56281-70339 | up | 7.50 | Beta-glucosidase 12 |
| TCONS_00041679 | 248881-259203 | up | 9.42 | L484_008768 | 207499-211649 | down | -4.24 | (-)-germacrene D synthase |
| TCONS_00019770 | 222314-428777 | down | -16.73 | L484_021156 | 426153-429613 | down | -5.31 | (-)-germacrene D synthase |
| TCONS_00019771 | 223650-359431 | down | -17.30 | L484_021156 | 426153-429613 | down | -5.31 | (-)-germacrene D synthase |
| TCONS_00019769 | 221908-428777 | down | -16.60 | L484_021156 | 426153-429613 | down | -5.31 | (-)-germacrene D synthase |
| TCONS_00019770 | 222314-428777 | down | -16.73 | L484_021147 | 220387-224291 | down | -3.54 | (-)-germacrene D synthase |
| TCONS_00019771 | 223650-359431 | down | -17.30 | L484_021147 | 220387-224291 | down | -3.54 | (-)-germacrene D synthase |
| TCONS_00019769 | 221908-428777 | down | -16.60 | L484_021147 | 220387-224291 | down | -3.54 | (-)-germacrene D synthase |
| TCONS_00081486 | 648289-658012 | down | -14.95 | L484_021932 | 593474-597091 | down | -2.52 | Jasmonate O-methyltransferase |
| TCONS_00025313 | 258904-259851 | down | -3.61 | L484_012855 | 341144-342339 | down | -8.56 | hypothetical protein L484_012855 |
| TCONS_00018797 | 134210-183709 | down | -14.51 | L484_006270 | 184802-192174 | down | -3.84 | hypothetical protein L484_006270 |
| TCONS_00018773 | 133607-182566 | down | -1.97 | L484_006270 | 184802-192174 | down | -3.84 | hypothetical protein L484_006270 |
| TCONS_00018772 | 123133-133181 | down | -12.61 | L484_006270 | 184802-192174 | down | -3.84 | hypothetical protein L484_006270 |
| TCONS_00018784 | 181064-182566 | down | -12.65 | L484_006270 | 184802-192174 | down | -3.84 | hypothetical protein L484_006270 |
| TCONS_00018805 | 180640-182563 | down | -8.03 | L484_006270 | 184802-192174 | down | -3.84 | hypothetical protein L484_006270 |
| TCONS_00007049 | 81307-83057 | down | -9.14 | XLOC_004630 | 83343-84427 | down | -9.76 | - |
| TCONS_00034213 | 28923-66130 | down | -13.45 | L484_003022 | 15520-16366 | down | -10.73 | hypothetical protein L484_003022 |
| TCONS_00034208 | 8312-8552 | down | -7.69 | L484_003022 | 15520-16366 | down | -10.73 | hypothetical protein L484_003022 |
| TCONS_00034229 | 50763-51139 | up | 8.12 | L484_003022 | 15520-16366 | down | -10.73 | hypothetical protein L484_003022 |
