# Supplementary material for: Long noncoding RNA transcriptome analysis reveals novel lncRNAs in Morus alba ‘Yu‐711’ response to drought stress
Source: Plant Genome. 2022 Oct 26;17(1):e20273. doi: 10.1002/tpg2.20273 (PMC12807294; doi:10.1002/tpg2.20273)
Supplement: Supplementary file 2 [file TPG2-17-e20273-s006.docx]

**Supplementary Materials For**

**Long non-coding RNA transcriptome analysis reveals novel lncRNAs in Morus alba variety Yu-711 response to drought stress**

Michael Ackah ^a, g #*^ Jin Xin ^a#^, Qiaonan Zhang ^a#^, Frank Kwarteng Amoako ^b^, Lei Wang ^a^, Thomas Attaribo ^c^, Mengdi Zhao ^d^, Feng Yuan ^a^, Richard Ansah Herman ^a^,
Changyu Qiu ^e^, Qiang Lin ^e^, Zhi Yin ^f^, Weiguo Zhao ^a, g *^

^a^ Jiangsu Key Laboratory of Sericultural Biology and Biotechnology, School of Biotechnology, Jiangsu University of Science and Technology, Zhenjiang 212100, China.
^b^ Institute of Plant Nutrition and Soil Science, Kiel University, Hermann-Rodewald-Straße 2, 24118 Kiel, Germany.
^c^ School of Agriculture, C. K. Tedam University of Technology and Applied Sciences, Navrongo UK-0215-5321, Ghana.
^d^ Department of Materials Science and Engineering, Suzhou University of Science and Technology, 99 Xuefu Road, Huqiu District, Suzhou 215004, China.
^e^ Sericultural Research Institute, Guangxi Zhuang Autonomous Region, Nanning 530007, China
^f^ Nanjing University of Finance & Economics, Nanjing 210023, China.
^g^ Key Laboratory of Silkworm and Mulberry Genetic Improvement, Ministry of Agricultural and Rural Affairs, Sericultural Research Institute, Chinese Academy of Agricultural Sciences, Zhenjiang, Jiangsu 212100, China

^#^ These authors contributed equally to the work

To whom all correspondence should be addressed.
^*^ Correspondence: [wgzsri@126.com](mailto:wgzsri@126.com); +8613775327390: [ackahmichael90@gmail.com](mailto:ackahmichael90@gmail.com); +8618921584239

**Supplementary materials**

**Figure S1**. Pearson correlation between EG and CK samples. EG1-EG2; drought stress treatment. CK1-CK2; control treatment.

**Figure S2.** Distribution of base content along with reads. Type A, T, G, and C are the individual nucleotide bases, and N is read containing unknown bases

**Figure S3.** Reads distribution in different regions of the genome.

**Figure S4.** Validation of candidate lncRNAs. 1 μg total RNA extracted from mulberry (Yu-711) leaves was reverse transcribed into cDNA. Specific primers for seven lncRNAs were used to perform PCR. The PCR products were separated on 1% agarose gel to detect the expression of lncRNAs. Ck. control; a. TCONS_00081189; b. TCONS_00059994; c. TCONS_00038679; d. TCONS_00055241; e. TCONS_00073297; f. TCONS_00090844; g. TCONS_00069106

**Figure S5**. Venn diagrams for two groups comparison of the differential transcripts expression patterns. (a) lncRNAs comparison in CK and EG. (b) mRNAs comparison in CK and EG. (c) unclassified transcripts comparison in CK and EG.

**Figure S6.** Principal component analysis (PCA) of the samples.

**Figure S7.** Predicted DElncRNAs-miRNAs and DEmRNAs target-miRNAs regulatory networks in response to drought stress. (a) DElncRNAs-miRNAs regulatory networks from the biosynthesis of secondary metabolites pathway. The Triangle shape indicates miRNAs, and the eclipse shape indicates DElncRNAs, red indicates up-regulation, and green indicates down-regulation. (b) DElmRNAs target-miRNAs regulatory networks. The Triangle shape indicates miRNAs, and the diamonds indicate DEmRNAs. Red color indicates up-regulation, and green color indicates down-regulation.

**Figure S8**. Phylogenetic heatmap of the transcripts validated by quantitative real-time PCR for the relative expression level of DEmRNA-target genes (a) and DElncRNA (b). The trees were constructed with MEGA 11 software using the Neighbour-Joining (NJ) method and Boostrap with 1000 replicates. TBtools software was used to build the heatmap.

**Figure S9**. Distribution of AS event in mulberry plant response to drought stress and control.

**Figure S10**. protein-protein interaction network in response to drought stress in mulberry leaves. The lines represent the edges, and the V-shape is the node

**Figure S1**. Pearson correlation between EG and CK samples. EG1-EG2; drought stress treatment. CK1-CK2; control treatment.

**Figure S2.** Distribution of bases content along with reads. Type A, T, G, and C are the individual nucleotide bases, and N is read containing unknown bases a,b; control samples. c,d; drought-stress samples,

**Figure S3.** Reads distribution in different regions of the genome. a,b; control samples. c,d; drought-stress samples

**Figure S4.** Validation of candidate lncRNAs. 1 μg total RNA extracted from mulberry (Yu-711) leaves was reverse transcribed into cDNA. Specific primers for seven lncRNAs were used to perform PCR. The PCR products were separated on 1% agarose gel to detect the expression of lncRNAs. Ck. control; a. TCONS_00081189; b. TCONS_00059994; c. TCONS_00038679; d. TCONS_00055241; e. TCONS_00073297; f. TCONS_00090844; g. TCONS_00069106

**Figure S5**. Venn diagrams for two groups comparison of the differential transcripts expression patterns. (a) lncRNAs comparison in CK and EG. (b) mRNAs comparison in CK and EG. (c) unclassified transcripts comparison in CK and EG.

**Figure S6.** Principal component analysis (PCA) of the samples

**Figure S7.** Predicted DElncRNAs-miRNAs and DEmRNAs target-miRNAs regulatory networks in response to drought stress. (a) DElncRNAs-miRNAs regulatory networks from the biosynthesis of secondary metabolites pathway. The Triangle shape indicates miRNAs, and the eclipse shape indicates DElncRNAs, red indicates up-regulation, and green indicates down-regulation. (b) DElmRNAs target-miRNAs regulatory networks. The Triangle shape indicates miRNAs, and the diamonds indicate DEmRNAs. Red color indicates up-regulation, and green color indicates down-regulation

**Figure S8**. Phylogenetic heatmap of the transcripts validated by quantitative real-time PCR for the relative expression level of DEmRNA-target genes (a) and DElncRNA (b). The trees were constructed with MEGA 11 software using the Neighbour-Joining (NJ) method and Boostrap with 1000 replicates. TBtools software was used to build the heatmap

**Figure S9**. Distribution of AS event in mulberry plant response to drought stress and control

**Figure S10**. protein-protein interaction network in response to drought stress in mulberry leaves. The lines represent the edges, and the V-shape is the node
